# Metabolic Classification and Intervention Opportunities for Tumor Energy Dysfunction

**DOI:** 10.3390/metabo11050264

**Published:** 2021-04-23

**Authors:** Ezequiel Monferrer, Isaac Vieco-Martí, Amparo López-Carrasco, Fernando Fariñas, Sergio Abanades, Luis de la Cruz-Merino, Rosa Noguera, Tomás Álvaro Naranjo

**Affiliations:** 1 Department of Pathology, Medical School, INCLIVA Biomedical Health Research Institute, University of Valencia, 46010 Valencia, Spain; emonferrer@incliva.es (E.M.); iviemar@alumni.uv.es (I.V.-M.); amparolopezcarrasco@gmail.com (A.L.-C.); 2 Low Prevalence Tumors, Centro de Investigación Biomédica en Red de Cáncer (CIBERONC), Instituto de Salud Carlos III, 28029 Madrid, Spain; 3 Ynmun Group, Institute of Clinical Immunology and Infectious Diseases, 29004 Málaga, Spain; farinas.inmunologia@gmail.com; 4 Integrative and Conscious Health Institute, 08008 Barcelona, Spain; s.abanades@gmail.com; 5 International College of Human Nutrition and Functional Medicine, 08001 Barcelona, Spain; 6 Clinical Oncology Department, Hospital Universitario Virgen Macarena, 41009 Sevilla, Spain; ldelacruzmerino@gmail.com; 7 Department of Pathology, Verge de la Cinta Hospital of Tortosa, Catalan Institute of Health, Institut d’Investigació Sanitària Pere Virgili (IISPV), 43500 Tortosa, Spain; 8 Department of Basic Medical Sciences, Medical School, Rovira i Virgili University, 43201 Reus, Spain

**Keywords:** tumor ecosystem, tumor reprogramming, tumor microenvironment

## Abstract

A comprehensive view of cell metabolism provides a new vision of cancer, conceptualized as tissue with cellular-altered metabolism and energetic dysfunction, which can shed light on pathophysiological mechanisms. Cancer is now considered a heterogeneous ecosystem, formed by tumor cells and the microenvironment, which is molecularly, phenotypically, and metabolically reprogrammable. A wealth of evidence confirms metabolic reprogramming activity as the minimum common denominator of cancer, grouping together a wide variety of aberrations that can affect any of the different metabolic pathways involved in cell physiology. This forms the basis for a new proposed classification of cancer according to the altered metabolic pathway(s) and degree of energy dysfunction. Enhanced understanding of the metabolic reprogramming pathways of fatty acids, amino acids, carbohydrates, hypoxia, and acidosis can bring about new therapeutic intervention possibilities from a metabolic perspective of cancer.

## 1. Introduction

The energy required for life is produced by cellular metabolism. Initiating metabolic pathways requires the participation of enzymes and mechanisms dependent on specific gene activation, which are inextricably linked to cellular metabolic and energetic activity. Metabolic alterations in tumor cells to provide increased energy function, known as metabolic reprogramming, facilitate proliferation, infiltrative capacity, distant growth, and treatment resistance, among other tumor activities. This is a crucial hallmark of tumorigenesis, underpinning the specific alterations of each tumor change, regardless of the biological perspective (morphological, biochemical, physical, immunophenotypic, molecular or genetic) adopted [1,2]. The metabolic profile of the tumor determines the biological properties of tumor cells, immune response, tumor microenvironment configuration, morphological and molecular tumor heterogeneity, and many other variables, including treatment response and prognosis [3,4]. Considering changes in metabolic pathways, tumor disease can be understood as a bioenergetic dysfunction, regardless of the organ involved, clinicopathological staging, histopathologic classification, or mutational/copy number aberration burden [5,6]. During the last century, energy dysfunction in cancer has been identified as a key mechanism in pathogenesis, and among relevant fields of study are nuclear transplantation effects, genetic and epigenetic adaptation of the tissue, biotensegral mechanisms, and the influence of the Warburg effect. Warburg’s hypothesis concerning the tendency of tumor cells to increase glucose consumption through fermentation has experienced a revival, and is now recognized as a common event in tumorigenesis [7]. The use of the glycolytic pathway in malignant transformation for adenosine triphosphate (ATP) production, regardless of oxygen availability, and its low energy efficiency, clearly points to tricarboxylic acid cycle (TCA) modification and mitochondrial shape and function alterations found in many tumors. Among the most compelling evidence for this phenomenon comes from diagnostic techniques, such as positron emission tomography (PET) [8], which have been used to describe an increase in metabolic alterations from benign to malignant tumors according to their clinical aggressiveness, metastasis, and treatment response [9,10]. Different preventive and therapeutic approaches related to oncological metabolic reprogramming are also emerging.

Proposals for tumor classification and tumor heterogeneity according to the degree of metabolic alteration are particularly interesting from a metabolic reprogramming point of view [11]. Indeed, recent data on hepatocarcinoma [12], prostate [13], and colon cancer [14] support classifying tumors according to their energy dysfunction degree, independently of the tumor tissue origin. An important biological consideration in cancer, tumor heterogeneity, understood as differing morphological, immunophenotypic, and genotypic profiles that can occur in different areas of the same tumor, between the primary tumor and its metastases (spatial and/or temporal intratumoral heterogeneity), and/or between different tumors (intertumoral heterogeneity), can also be explained by varying degrees of energy dysfunction [15]. Tumor heterogeneity, which also contributes to determining tumor classification, is related to tumor cell proliferation and differentiation, tumor microenvironment modification, treatment response and resistance acquisition, and patient survival [16,17,18,19].

Metabolic reprogramming [5] and new therapeutic approaches aimed at recovering altered lipogenesis, glutaminolysis, or glycolysis pathways [6] have prompted use of a metabolic and bioenergetic medicine approach as a powerful new alignment tactic in cancer. The main objectives of this review are to summarize the metabolic pathways involved in cancer energy dysfunction, devise a classification proposal based on this, and highlight avenues of therapeutic intervention from a metabolic perspective of cancer.

## 2. Evidence of Changes in the Metabolic Pathways of Tumor Energy Dysfunction

Different mechanisms involved in cell biology, such as fat, protein, or carbohydrate pathways, as well as pH regulation and oxygenation, have been found to be altered in different tumors.

### 2.1. Tumors with Transformed Lipid Metabolism Pathways

**Figure 1:**
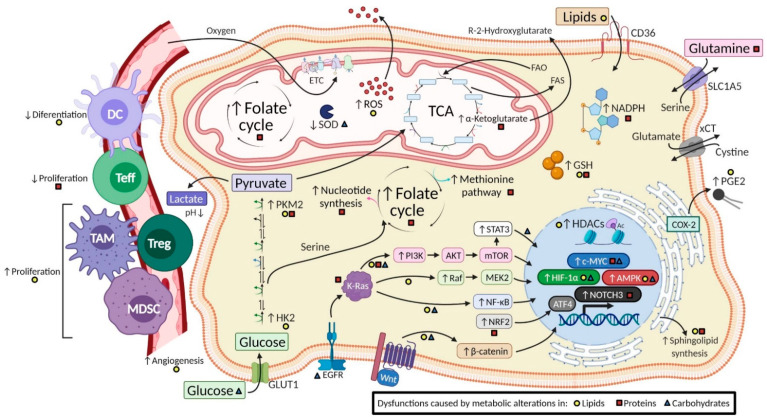
Summary of alterations of metabolic pathways in tumor cells and subsequent changes in cellular processes and the tumor microenvironment. The diagram shows a selection of the tumor metabolic alterations in lipids, proteins, and carbohydrates described in the text. Some subsequent regulations caused by metabolic modifications within the cell are highlighted, such as changes in molecular pathways, transcription factors, and biomolecules and in the tumor microenvironment caused by immune cells, blood vessels, and changes in pH. The yellow circle, red square, and blue triangle indicate that the change is promoted by metabolic alteration in lipids, proteins, and carbohydrates, respectively. The schema is based on data from the literature review. Certain cellular and microenvironmental tumor alterations can also be attributed to other metabolic alterations not mentioned in this review. DC: Dendritic cells; ETC: Electron Transport Chain; FAO: Fatty Acid Oxidation; FAS: Fatty Acid Synthesis; GSH: Glutathione; HDAC: Histone Deacetylase; HK2: Hexokinase 2; MDSC: Myeloid-Derived Suppressor Cell; PEG2: Prostaglandin E2; PKM2: Pyruvate Kinase M2; ROS: Reactive Oxygen Spices; SOD: Super Oxide Dismutase; TAM: Tumor Associated Macrophages; TCA: Tricarboxylic Acid Cycle; Teff: T effector cells; Treg: T regulatory cells.

Lipids are central actors in cancer biology, displaying an essential structural role at the membrane level, acting as energy fuel and playing a key role as signaling and regulating molecules of cellular functions [20]. Both tumor cells and non-tumor cells reconfigure their metabolism substantially and establish specific lipid profiles recognizable as biomarkers with diagnostic, prognostic, and predictive potential. Table 1 summarizes lipid metabolism-targeted pathways, upregulated and downregulated biomarkers, tumor types affected, and therapeutic agents reported. Figure 1 schematizes altered lipid pathways associated with cancer.

Although tumor cells usually biosynthesize fatty acids through fatty acid synthase (FASN) [56] rather than acquiring them from the diet, in adipocyte-rich tissues, such as bone marrow, tumor cells rely on exogenous lipids to regulate cellular energetics and adapt to harsh metabolic conditions of the metastatic niche [21]. FASN has been described as increased in cancer patients [57] and correlated with clinical behavior and aggressiveness in different tumors, such as ovarian, breast [25], prostate [27], pancreas, and colon [30]. Recent studies have focused on the synergistic effect of different compounds seeking both anticatabolic and antianabolic action [32]. FASN inhibitors, such as cerulenin and orlistat, induce apoptosis and delay tumor growth [33], and an analogous effect can be obtained from natural sources, such as green tea and soybeans. Finally, it has been reported that proton pump inhibitors can inhibit FASN and extend survival in breast cancer patients [26,34]; however, precaution must be taken concerning its continued use in the population, since an increased risk of pancreatic cancer has also been associated with its use [31]. Omega-3 and omega-6 polyunsaturated fatty acids (PUFAs) act as signaling molecules for immune processes in numerous inflammatory diseases and in solid and hematological tumors [37,41]. Furthermore, recent studies have recognized the important role of PUFAs in tumor response to immunotherapy [29].

Other cellular energy production inhibitors from fatty acid oxidation (FAO) pathways have shown experimental clinical efficacy in solid tumors and leukemia [35,36]. In healthy tissue, the change from anabolism to catabolism to halt proliferation implies decreased glycolysis and higher fatty acid oxidation, as regulated by the Randle cycle. Some tumors require a simultaneous increase in mitochondrial fatty acid oxidation and glycolysis to support anabolism and proliferation, thus escaping the competitive nature of the Randle cycle [58]. Mitochondrial metabolic reprogramming blockade has been used therapeutically to selectively arrest tumor growth [58,59].

Prostaglandins derived from arachidonic acid possess oncogenic functions. Their synthesis enzymes are overexpressed in different tumors and offer a new therapeutic target for tumors that show alterations in this metabolic pathway [38,39]. Inflammatory mediators, such as prostaglandin E2, are not only implicated in tumor aggressiveness and tumor microenvironment changes, but also unify the pathophysiology of many other different diseases, such as neurodegenerative, metabolic, and autoimmune disorders [40]. In fact, inflammatory mediators alter mechanisms common to all these, such as apoptosis escape, growth factor receptor activation, angiogenesis induction, and immune regulation.

The critical functions of bioactive sphingolipids are present in most biological responses, cell signaling, and normal and pathological metabolism [60], including those seen in cancer [44]. They have been reported to promote malignant transformation and tumor progression in tumor energetic dysfunction [22]. Sphingomyelin can be converted by sphingomyelinase to ceramide and from this to sphingosine-1-phosphate (S1P) [61]. All derivatives have been considered to participate in tumor progression, being differently regulated in multiple solid carcinomas [46], especially digestive and prostate ones [43,51] and leukemia [52]. Approaches for ceramidase inhibition have been developed from these observations, obtaining results, such as cancer cell apoptosis, tumor growth delay [45], decreased tumor angiogenesis [49], increased sensitization to chemotherapeutic agents [50], and resistance treatments [62]. Similarly, dietary supplementation with sphingomyelin experimentally reduces intestinal tumor development [54,55]. Moreover, a novel S1P receptor modulator, fingolimod, has clinical applications in autoimmune, inflammatory, and tumor diseases [47]. Studying its action at the microenvironment level, with a focus on the extracellular matrix, angiogenesis and macrophage infiltration in multiple sclerosis, ulcerative colitis, and colon cancer [48], is of particular interest from an essential energy dysfunction standpoint.

It is known that lipid metabolic abnormalities in cancer, such as increased fatty acid oxidation and de novo lipid synthesis, provide resistance to chemotherapy, radiotherapy, and other survival advantages for tumor cells through their multiple effects on the tumor microenvironment [23]. There is thus significant potential for therapeutic intervention and modulation based on altered metabolic pathways and lipid signaling when present in tumors and their microenvironments, as has been described in breast and bone cancer and other tumors [22].

### 2.2. Tumors with Altered Amino Acid Metabolism

Several tumors metabolize amino acids: to meet their own demands, to adjust the tumor microenvironment structure and function, or to generate resistance to conventional chemotherapy treatments. Recently, amino acid depletion therapies targeting amino acid uptake and catabolism have been employed, using heterologous enzymes or recombinant or modified human enzymes [63]. These therapies have little effect on normal cells due to their lower amino acid demand but exert intense action on tumor cells with high proliferative and biosynthetic demand for key amino acids. A summary of the protein metabolism targeted pathways, upregulated and downregulated biomarkers, tumor types affected, and reported therapeutic agents can be seen in Table 2. Figure 1 schematizes alterations of amino acid pathways associated with cancer.

Serine and glycine have been found to support oncogenic stimulus in a group of tumors [67,68]. Their role is critical in one-carbon (1C) metabolism [71], a vital network for different oncogenic mediators. The 1C units are produced inside the mitochondria and exported to the cytoplasm, where they are used to produce glycine and NADPH [76]. One-carbon metabolism is mediated by the folate cofactor, which regulates purines and thymidine synthesis, amino acid homeostasis (glycine, serine, and methionine), epigenetic modulation, redox protection, and many other physiological processes. Since these amino acids and their synthesis enzymes are highly present in different tumors, specific strategies have been proposed to block their synthesis, especially for serine [77]. Several lung cancer types activate an NRF2-dependent transcriptional program that regulates serine and glycine metabolism, especially in the initiating stage [70], and is related to clinical aggressiveness [65]. Additionally, NRF2-positive regulation is also associated with chemotherapy and radiotherapy resistance [72]. Furthermore, highly proliferating cells (tumor and antitumor immune cells) depend on serine since it participates in the anabolism of multiple substances through the 1C metabolism. Therefore, serine metabolism unifies different diseases under a common pathophysiological pathway, providing new preventive and therapeutic approaches. Other protein pathway changes occur in digestive system [66], breast [74], hematological [75], and other tumors, which is of special interest in potential specific metabolic treatments [69].

Glutamine metabolism regulation has emerged as a potential antitumor therapeutic approach [79,80] due to its key role in glutathione formation and TCA activity. The viability and energy resources of several tumor types have been shown to be dependent on this pathway, so drugs able to influence this pathway seem promising specific treatments in a wide group of tumors [83]. Inhibitors of glutaminase (GLS), the enzyme that converts glutamine to glutamate, and glutamine transport inhibitors are one focus of research. Pharmacological inhibition of GLS alone or in combination with immune checkpoint blockade represents an effective therapeutic strategy for cancers involving alterations in the SWI/SNF complex, which occurs in more than 60% of clear cell ovarian carcinoma, which otherwise has no effective treatment [81]. GLS knockdown, exposure to the GLS inhibitors, or deprivation of glutamine resulted in robust induction of reactive oxygen species in GLS-expressing ovarian cancer cells in one study, and treatment with GLS inhibitor could effectively treat chemoresistant ovarian cancers, especially those with high GLS expression [82].

Asparagine is also involved in malignant transformation and tumor progression [87]. It is converted by asparaginase enzyme to aspartic acid, which is related to treatment resistance in acute lymphoblastic leukemia [84], while asparagine synthetase (ASNS), which catalyzes the reverse conversion, may also be a therapeutic target [86]. A significant proportion of lung and colon cancer tumors possess mutated *KRAS*, which regulates asparagine biosynthesis and alters sensitivity to L-asparaginase. *KRAS* mutation causes a marked decrease in aspartate levels and increases asparagine levels in which ASNS expression is upregulated and induced by the KRAS-activated signaling pathway. KRAS-mutant cancer cells could become adaptive to glutamine depletion through asparagine biosynthesis by ASNS; pronounced growth suppression was observed upon ASNS knockdown, indicating that ASNS might be a novel therapeutic target against tumors with mutated *KRAS* [85].

### 2.3. Tumors with Carbohydrate Pathway Modifications

Given their energy impact, carbohydrate pathways are the most shared metabolic alterations in tumors [88]. A summary of the carbohydrate metabolism targeted pathways, upregulated and downregulated biomarkers, tumors affected, and reported therapeutic agents can be seen in Table 3. Figure 1 depicts the carbohydrate pathway alterations associated with cancer.

Otto Warburg’s abovementioned discovery of high rates of aerobic glycolysis in cancer cells suggested structural and/or functional impairment of the oxidative phosphorylation process (OXPHOS). Interestingly, different morphological tumors, such as oral squamous cell carcinoma [89], lung [90], breast [91], pancreatic carcinomas [92], and hepatocarcinoma [93], show various alterations in carbohydrate metabolic pathways. In normal cells, a high glycolysis rate is linked to a reduction in OXPHOS, known as the Crabtree effect [94]. Unless tumor cells become hypoxic, they maintain high glycolysis and OXPHOS rates to meet the high energy demand of anabolic processes. However, these main pathways are interconnected with other pathways that also require glucose, including the pentose phosphate pathway, which produces pentose phosphates for ribonucleotide and NADPH synthesis; the hexosamine pathway, for glycosylation of proteins; glycogenesis, which generates glycogen for glucose storage; the serine biosynthesis pathway, which generates amino acids; and 1C metabolism, which generates NADPH, purine and glutathione biosynthesis, and methylation [88].

The extent of carbohydrate metabolic alteration can determine the tissue injury type, and whether it corresponds to benign or malignant proliferative reactive injury, such as overexpression of hexokinase 1 (HK1) in colorectal carcinoma, which appears as an independent prognostic factor [95]. Interestingly, hexokinase 2 (HK2), which is required for anaerobic glycolysis, is frequently overexpressed in several malignant cells. HK2 expression increases progressively from glottis polypus to papilloma or laryngeal squamous cell carcinoma as the clinical aggressiveness of the tumor increases [9]. HK2 overexpression is also correlated with prognosis in tumors of the digestive system, including stomach, liver, pancreas, colon, and rectum [96], and breast cancer metastasis [97]. Moreover, HK2 has been confirmed as an independent prognostic indicator in advanced cervical squamous cell carcinoma, and its expression has been correlated with the degree of radiation resistance [98]. Likewise, it has been documented that higher HK2 expression correlates with cisplatin chemoresistance in ovarian cancer [99].

### 2.4. Dysregulated pH as a Hallmark of Cancer

Since low pH is characteristic of malignant tumors and relates to tumor treatment resistance, antacids, such as proton pump inhibitors, have been proposed to improve chemotherapy results [34]. Nevertheless, this is an emerging field with heterogeneous results, which urges caution [114]. Several studies have been conducted on the regulation of membrane transporters, electrolyte exchangers, enzymes, water trafficking, modifications of membrane structure, transcription factor deviation, metabolic changes, and many other effects of lactate accumulation due to tumor metabolic alteration [115]. Further studies in this field will enhance our understanding of common pathophysiological mechanisms shared by different diseases, such as metabolic disorders and malignant tumors [116].

### 2.5. Tumors with Hypoxic Adaptation

Solid tumors present low oxygenation levels, which results in proliferative stimulus, extensive tumor infiltration, and metabolic reprogramming, mediated by hypoxia-inducible factors (HIFs) [117]. HIF-1α controls the expression of numerous genes encoding metabolic enzymes, which play key roles in cellular metabolism adaptation to low oxygen tension [118]. An interesting mechanism is the positive feedback loop between HIF-1α and the nicotinamide phosphoribosyltransferase (NAMPT), which is the first and rate-limiting enzyme of the route that recycles the nicotinamide adenine dinucleotide (NAD) [119]. NAMPT can be found intracellularly (iNAMPT), where it initiates the synthesis of the NAD, necessary to maintain metabolic processes, such as glycolytic flux and lactate production. Furthermore, NAMPT can also be found extracellularly (eNAMPT), where it has cytokine-like functions (through Toll-like receptor 4), promoting the differentiation of TAMs and MDSCs [119,120]. Under hypoxic conditions, upregulation of iNAMPT/eNAMPT promotes metabolic reprograming and an immunosuppressive microenvironment, and several data show that this alteration can play a central role in the phenotypic plasticity of melanomas [119,120]. In addition, HIF-1α also mediates the serine synthesis pathway and 1C mitochondrial metabolism to increase production of mitochondrial antioxidants (NADPH and glutathione), which opens up interesting possibilities for therapeutic intervention in the microenvironment and tumor resistance [1,121]. Additionally, hypoxia promotes treatment resistance and tumor progression, altering glucose and amino acid absorption, glycolytic flow, lactate production, glutamine metabolism, modifying the TCA cycle and OXPHOS process, and fatty acid synthesis, and generating high levels of mitochondrial reactive oxygen species (ROS) [122].

## 3. Intervention Opportunities from a Metabolic View of Cancer

Developing new therapies based on specific metabolic reprogramming requires studying tumor bioenergetic dysfunction, by enabling rapid and easy detection of specific biomarkers for each altered metabolic pathway. In this section, we review the therapeutic protocols established for this purpose, which can be complemented with nutritional approaches and action on macroenvironment factors (exercise, diet, microbiota, or stress). Table 1, Table 2 and Table 3 summarize avenues of therapeutic intervention reported from a metabolic tumor reprogramming perspective.

### 3.1. Intervention on the Fatty Acid Pathway

Since changes in lipid metabolism in cancer cells affect numerous cellular processes, including cell growth, proliferation, differentiation, and survival, several enzymes and regulatory factors involved in these pathways have come to light as targets in tumor treatment [123].

Although the efficacy of statins as lipid-lowering agents is still under study, their capacity to reduce cancer death risk and increase cancer patient survival has already been reported [124]. Their action mechanism at the mitochondrial level involves factors such as increased tumor cell radiation sensitivity [125]. Statins also contribute to increasing autophagy, and when combined with metformin have an apoptosis-inducing effect even in chemoresistant cells [126].

Likewise, the putative beneficial effect of omega-3 dietary supplementation during cancer treatment has been accentuated [127]. Some studies suggest that this nutritional complement may reduce inflammation and cytolytic treatment toxicity, and enhance chemotherapy efficacy. Furthermore, omega-3 supplementation can be used in cancer cachexia treatment due to its positive role in maintaining patient weight [42].

### 3.2. Intervention in the Protein Pathway

Amino acids from the diet are essential for tumor cell proliferation and survival. Serine intake restriction affects cell proliferation and mitochondrial function [78] as well as carbohydrate and fatty acid metabolic pathways. The same occurs with glutamine, a multifunctional amino acid involved in lipid metabolism, energy balance, apoptosis, and cell proliferation, modifying several proteins that depend on its availability in both normal [128] and tumor cells [129].

Available data suggest that a low-protein diet could be advantageous for cancer patients [130]. Dietary restriction of serine and glycine can reduce tumor growth and increase survival in some carcinoma and lymphoma models [131]. Exogenous amino acid availability can also be reduced by blocking their transporters [69] as a viable strategy to reduce tumor growth [132] and therapeutic resistance [133]. Additionally, preclinical experiments suggest that a short fasting period before radiation and/or a transient caloric restriction during the treatment course can increase tumor responsiveness [134]. This mechanism works by promoting accumulation of cellular oxidative injury, hampering DNA repair, and stimulating tumor cell death. Healthy cells have a more flexible metabolism, which allows them to activate repair and survival mechanisms. Furthermore, the immune system responds by stimulating an effective antitumor response, especially in tumors with high glucose uptake detected by PET [134].

### 3.3. Intervention in the Carbohydrate Pathway

Metabolic therapies that decrease circulating glucose levels slow the progression of several tumors. Following on from this, metformin [135] and aerobic glycolysis regulatory compounds, such as 2-deoxyglucose, oxythiamine, and 6-aminonicotinamide, could be viewed as novel antitumor metabolic therapies [136].

Metformin is an antidiabetic agent with a powerful epigenetic effect, able to impact directly on cancer cell proliferation by altering DNA methylation [137], and which has shown an anticancer effect [110]. Its action mechanism includes decreasing blood glucose levels [138] and glycolytic flux, suppressing HIF-1α expression [139], and interfering with cancer stem cell functions. Furthermore, it regulates stromal vascularization, facilitating metastasis suppression and enhancing chemotherapy’s effect [140], and induces apoptosis and autophagy. Other therapeutic approaches that inhibit tumor growth are glucose transport inhibitors, such as phloretin, and mitochondrial oxidative metabolism stimulators, such as frataxin [141]. Moreover, well-known therapeutic agents, such as imatinib and trastuzumab, also target signaling pathways linked to glucose metabolism [100]. The Warburg effect can be targeted with dichloroacetate, which induces apoptosis only in cancer cells [111], and arsenic trioxide, which increases mitochondrial glutaminolysis activity. By detecting specific metabolic alterations present in both the original tumors and those resistant to conventional treatments, complementary measures can be included to reduce glucose availability, either with specific glycolysis inhibitors or through nutritional approaches. Increased therapy responses have recently been reported when HK2 was inhibited with 3-bromopyruvate in hepatocarcinoma [142] and breast cancer [143], with resveratrol or sinomenine in lung cancer [106], with lonidamine in cholangiocarcinoma [144], and with luteolin in gastric cancer [145].

A complementary strategy has been proposed for when conventional therapeutic agents or supplementation are not safe or available, based on carbohydrate intake reduction, blood glucose level monitoring, and the application of clinical knowledge and research from diabetes and other metabolic diseases treatment [146]. Low-carb natural interventions aimed at avoiding carbotoxicity include ketogenic diets [147] and various fasting modalities [101]. Recent studies show good tolerance, safety, and beneficial clinical effects, which make the case for adding these complementary measures to conventional chemotherapy in comprehensive oncology treatment [103].

### 3.4. Chemotherapy and the Fundamental Role of Immunotherapy

Other metabolic pathways are targeted in classic cancer treatments. A frequently used pyrimidine analog called 5-Fluorouracil, employed particularly in colorectal cancer, targets nucleotide metabolism derived from the folate cycle [107]. Gemcitabine is used especially to treat pancreatic cancer: it interferes with cytidine biosynthesis and prevents deoxynucleotide formation. Other pharmacological agents, such as etoposide, doxorubicin cisplatin, and cyclosporine A, have shown their efficacy through interaction with nucleic acid metabolism [108]. It should be noted that resistance to several of these treatments depends on alteration of metabolic pathways, as occurs with cisplatin in chronic myeloid leukemia [148].

Metabolic reprogramming of the tumor immune response, given the different metabolites participating in the humoral and cellular immune response, establishes the pro- and anti-inflammatory balance, determines the activation process of effector T lymphocytes and other immune cell subpopulations, and is among the main regulatory mechanisms of immune checkpoints [149]. Immunotherapy is one of the clearest exponents of the relationship between cancer, metabolism, energy dysfunction, and immune response. Given that, to date, immunotherapy efficacy is limited to a fraction of patients, a deeper understanding is required of the mechanisms that generate an immunosuppressive tumor microenvironment, emanating from inappropriate metabolic reprogramming, which dampens T cell function and affects the antitumor immune response and tumor progression [150]. Immune and tumor cells use similar metabolic reprogramming. Once lymphocytes have been activated, they begin a metabolic transition from oxidative phosphorylation to aerobic glycolysis. Immune and tumor cells compete within the tumor microenvironment, and increased nutrient consumption by tumor cells achieves an immunosuppressive microenvironment by hindering T cell metabolism [151]; hence, interest in targeting both tumor and T cell metabolism exists, which can enhance immunity and improve the success of immunotherapies [152].

Upregulated programmed death-ligand 1 (PD-L1) and cytotoxic T-lymphocyte sntigen 4 (CTLA-4) alter T cells’ metabolic program, leading to their exhaustion [149]. The role of PD-L1 in cancer metabolic reprogramming comes from the balance between glycolytic activity in tumor cells and the available energy for cytotoxic T lymphocytes, which regulate tumor growth [153]. Overexpression in tumor cells of the glycolysis enzyme HK2 suppresses glucose uptake and interferon gamma (IFN-γ) production in tumor-infiltrating lymphocytes (TILs). Anti-PD-L1 treatment regulates glycolysis, increases antitumor immunity, and avoids the oncogenic effect of PD-L1 by inducing metabolic reprogramming and immune checkpoint. Glucose is a critical substrate for effector T cells and M1 macrophages, which both require aerobic glycolysis for their activation and full antitumor effector functions. Immunometabolism is therefore highlighted as a promising field in energy dysfunction treatment and tumor macroenvironment reprogramming [151]. Remarkably, immunotherapy action interconnects metabolic and immune regulation, diet [154], physical exercise, sleep, microbiota [155], and age [156], making these fundamentals of the macroenvironment a vital companion in comprehensive treatment of tumor energetic dysfunction.

### 3.5. Critical Role of the Tumor Microenvironment

A close relationship has been established between inflammation, carcinogenesis, and cancer therapy, which can trigger major changes in tumor stroma, including alteration of functional metabolic pathways contributing to cancer progression. An inflamed, acidified, and hypoxic tumor microenvironment triggers the activation of multiple gene and metabolic pathways that regulate cell survival, proliferation, and growth [157]. Increasing ROS production in tumors by HIF-1α, cytokines, and growth factors during hypoxic exposure activates survival pathways through their stromal and immune effect [158]. Metabolism and thus tumor behavior are regulated by metabolite availability in the tumor microenvironment [159]. As a metabolic niche, the tumor microenvironment is shaped by intrinsic tumor cell metabolism, interactions between tumor and non-tumor cells, and systemic metabolism [2,160]. The metabolic modification produced by lactate accumulation in the extracellular matrix acts as a resistance mechanism related to OXPHOS mitochondrial dysfunction. The relationship between ROS, metabolic reprogramming of cancer and stromal cells, and the transcription machinery involved in a malignant phenotype [158] indicates a possibility for new therapeutic management.

The clearly demonstrated plasticity capacity of tumor cells and their microenvironment represents an interesting option in the search for less toxic treatments, including healthy diet, physical exercise, and other lifestyle elements, such as tumor energy dysfunction modulators [161]. Dietary interventions impact on both cancer metabolism and immunometabolism [162] and changes in diet can improve cancer immunosurveillance and enhance the chemo-, radio-, and immunotherapy effect. The effect of these measures on cancer progression and overall survival [163], long-term survival [164] and recurrence [165], and their applicability across several life stages [166] makes these basic energy modulators promising complementary approaches to prevent, treat, and follow-up tumor energy dysfunctions. These measures are also affordable, cheap, and natural [167]. Clinical trials are needed to test the effectiveness of dietary interventions, such as caloric restriction or fasting [168] and supplementation with vitamin C [169], vitamin D [170], fermented foods, or probiotics [171], to regulate energy dysfunction, as well as microenvironment, stroma, and epigenetic reprogramming [172] in cancer and other diseases.

Therefore, remodeling ecosystem elements affected by energy dysfunction has emerged as an important therapeutic objective. In fact, the tumor microenvironment contributes decisively to the morphological, phenotypic, and genetic intratumoral heterogeneity that affects disease progression and promotes therapeutic failure; this has led to interest in focusing metabolic reprogramming measures both on tumor cellularity itself and on the tumor microenvironment.

## 4. Conclusions

Studies focused on metabolism underline tumor energy dysfunction, and cancers have shown to be appropriate models to identify the specific mechanism that underlies the growth, survival, mobility, and aggressiveness of tumor cells. The metabolic frame of reference considers oncological diseases as a diverse group of dysregulations, like in neurodegenerative or autoimmune illnesses. Cancer with fatty acid pathway changes will show different clinical-biological characteristics to others with altered protein or carbohydrate metabolism, despite all being recognized as oncological diseases regardless of organ or mutational or copy number aberration burden, depending on the degree of metabolic and energy dysfunction. Recent studies on hepatocarcinoma [12], prostate [13], and colon cancer [14] suggest classifying tumors according to their energy dysfunction degree. The group showing high metabolic activity tends to be associated with better prognosis, while those that show low metabolic activity have worse prognosis but high immune response, thus being more receptive to chemotherapy and immunotherapy [152]. The group with intermediate metabolic activity shows clinical behavior falling between the two previous groups. Patients with poor prognosis and more metabolically altered tumors with worse behavior, less response to treatment, and great tendency to chemotherapy resistance emerge as the greatest beneficiaries of tumor energy dysfunction reprogramming. Therapeutic regulation of the metabolic pathways of fatty acids, glutamine, or glucose requires previous characterization of genetic or metabolic biomarkers related to metabolic signatures. Generally, high glucose levels seem to contribute to cancer growth [102], while fats suppress metastasis [160].

The question arises as to whether alterations in the specific metabolic pathways of a tumor, organ, and patient occur before or after the genetic changes to which they are linked. Recent studies based on nuclear transfection experiments [173], epigenetic changes, and metabolism deviations (e.g., pathways, Warburg effect, pH, and oxygenation) support the idea that alterations in cellular bioenergetic mechanisms trigger biophysicochemical tissue changes that we identify as cancer. Beyond the tumor cell itself and its aberrations, the tumor microenvironment regulates nutrition, metabolism, and oncometabolic interaction with the host immune response [174], and factors, such as nutrition, stress, and microbiota [175], are all involved in global energy function. Metabolic heterogeneity plays a role in genetic heterogeneity [176], metastatic capacity of tumor cells [4], stem cells, and determining metastasis organotropism [177]. High tissue metabolic heterogeneity is frequently observed in different tumors. Metabolic heterogeneity determines treatment response, resistance acquisition, and metastasis prevention, making it an important consideration in cancer therapeutic approaches.

In summary, our current understanding of the different altered metabolic pathways involved in cancer energy dysfunction allows us to examine this disease from a new and different prism. It invites a classification of this disease according to the altered metabolic pathways and degree of bioenergetic dysfunction. The approach opens a new path of research and knowledge oriented at testing out new therapies based on metabolic reprogramming, plasticity, heterogeneity, and the proven possibility of tumor microenvironment remodeling. It enables study of the effect of already known antitumor drugs and their synergies with a demonstrated effect beyond cytoxicity, to further explore the promising field of immunotherapy and systematically include natural metabolic bioregulators, including diet, fasting, exercise, and control of microbiota and stress. Above all, through this approach, we can incorporate new intervention opportunities based on reprogramming of altered metabolic pathways, which must be studied in depth, beyond the data provided in this review, and identified with the appropriate biomarkers, to serve as a guide for tumor microenvironment remodeling of cancer understood as a reversible energetic dysfunction.

## Figures and Tables

**Table 1:** Lipid metabolic alterations in cancer.

Targeted Pathway	Biomarkers	Tumor Types Affected	Reported Therapeutic Agents
Cancer-associated adipose tissue Extracellular lipid uptake	**Tumor** [21,22,23]: CD36 increase FABP4 increase LPL increase **Adipocyte** [21,24]: ATGL increase HSL increase	Breast adenocarcinoma [21] Leukemia [21] Multiple myeloma [21] Prostate adenocarcinoma [21] Ovarian adenocarcinoma [21] Gastric adenocarcinoma [22] Pancreatic adenocarcinoma [22] Small cell lung cancer [22] Squamous cell carcinoma [22]	3-Bromopyruvate [21] CD36 inhibitors [22]
Fatty acid synthesis	FASN [25,26] SREBP1 [27,28] LXR [29] SCD-1 [29]	Breast adenocarcinoma [25] Colon adenocarcinoma [28,30] Pancreatic adenocarcinoma [26,31] Ovarian carcinoma [28] Prostate adenocarcinoma [27]	TVB-3136 [25] TVB-2640 [25] IPI-9119 [27] Cerulenin [28] Orlistat [28,32,33] C93 [28] Proton pump inhibitors [26,34]
Fatty acid oxidation	CPT1 [35] IDH2 [36]	Glioblastoma [36] Acute myeloid leukemia [35]	Etomoxir [35,36]
Prostaglandin E2	COX-2 [37,38] mPGES-1 [37] ID1 [38] ARC [37] EP4 [39]	Glioblastoma [38] Acute myeloid leukemia [37] Colorectal adenocarcinoma [39]	Prostaglandine receptors inhibitors [37,39,40] Omega-3 PUFA [37,41,42] Nonsteroidal anti-inflammatory drugs [39]
Bioactive sphingolipids	S1P increase [43] Ceramide decrease [43] Neutral ceramidase [43] Acid ceramidase [44,45] Sphk1 [44,46,47,48] S1PR1 [49] S1PR3 [49]	Colorectal adenocarcinoma [43,50] Prostate adenocarcinoma [44,51] Breast adenocarcinoma [44,46] Head and neck squamous carcinoma [44] Ovarian adenocarcinoma [44] Uterus adenocarcinoma [44] Acute myeloid leukemia [52] Glioblastoma [53]	C6 urea-ceramide [43] Dietary sphingomyelin [44,54,55] LCL385 [44] Fingolimod (FTY720) [44] L-t-C6-Pyr-Cer [44] LCL204 [52] Ceranib-b2 [49]

**Table 2:** Protein metabolic alterations in cancer.

Targeted Pathway	Biomarkers	Tumor Type Affected	Reported Therapeutic Agents
Serine Glycine	PHGDH [64,65] PSAT1 [65,66] PSPH [67,68] SLC1A4(ASCT-1) [67] SLC1A5(ASCT-2) [67,69] SHMT1 [67] SHMT2 [64,67] NFR2 [70,71,72,73,74,75]	Melanoma [68] Breast adenocarcinoma [67,74,76] Acute myeloid leukemia [75,76] Mesothelioma [64] Lung adenocarcinoma [67] Non-small cell lung cancer [65] Lymphomas [76] Colorectal adenocarcinoma [66]	Methotrexate [76,77] Pemetrexed [76] NCT-503 [77] Serine/glycine deprivation [68,78] Sulfonamide sulfonic ester scaffolds [69]
Glutamine Glutamate	xCT [79] SLC7A5/SLC3A2 [79] GLS1/2 [80,81,82] SLC1A5(ASCT-2) [69,80]	KRAS-driven cancer cells [79,81,82]	Glutamine deprivation [79] Aminooxyacetate (AOA) [79] CB-839 [80] BPTES [80] Sulfasalazine [80] V-9302 [80] Compound-968 [83] Sulfonamide sulfonic ester scaffolds [69]
Asparagine	ASNS [68,84,85] SLC1A5(ASCT-2) [69,86,87]	Acute lymphoblastic leukemia [68,84]	Adenylated sulfoximine 1 [84] Asparaginase [68] Sulfonamide sulfonic ester scaffolds [69]

**Table 3:** Carbohydrate metabolic alterations in cancer.

Targeted Pathway	Biomarkers	Tumors Affected	Reported Therapeutic Agents
Glucose uptake	GLUT1 [88,89,90,91,92]	Hepatocellular carcinoma [93] Renal cell carcinoma [88] Oral squamous cell carcinoma [89] Non-small cell lung cancer [90] Breast adenocarcinoma [91] Pancreatic adenocarcinoma [92]	Neoadjuvant chemoradiotherapy [92] Trastuzumab [100] Fasting [101] Fasting mimicking diet [101,102] Calorie restriction [103]
Glycolysis and TCA	HK1 [95] HK2 [9,93,96,97,98,99,104,105,106] PFKFB3 [88] PFK1 [88] PKM2 [107,108,109] IDH1 [110] PDK [111]	Laryngeal squamous cell carcinoma [9] Cervical squamous cell carcinoma [98] Hepatocellular carcinoma [96,105] Endometrial cancer [110] Breast adenocarcinoma [97,112] Epithelial ovarian cancer [99,111] Non-small cell lung cancer [106] Colon adenocarcinoma [107] Chronic myeloid leukemia [109]	Metformin [110] 2-Deoxyglucose [109] 3-Bromopyruvate [105] Increased frataxin [112] Dichloroacetate [111] Resveratrol [104] Sinomenine [106] Cyclosporine A [108] Overexpression of miR-122 [107] Overexpression of miR-202 [109]
Lactate production/extraction	LDHA [88,100] MCT1 [88] MCT4 [88]	Breast adenocarcinoma [100]	Metformin [113] Trastuzumab [100] Oxamate [100]
